# Treatment of Acute Myeloid Leukemia in Adolescent and Young Adult Patients

**DOI:** 10.3390/jcm4030441

**Published:** 2015-03-11

**Authors:** Guldane Cengiz Seval, Muhit Ozcan

**Affiliations:** Department of Hematology, Ankara University School of Medicine, Ankara 06100, Turkey; E-Mail: guldanecengiz@gmail.com

**Keywords:** acute myeloid leukemia, adolescent and young adults, treatment

## Abstract

The objectives of this review were to discuss standard and investigational treatment strategies for adolescent and young adult with acute myeloid leukemia, excluding acute promyelocytic leukemia. Acute myeloid leukemia (AML) in adolescent and young adult patients (AYAs) may need a different type of therapy than those currently used in children and older patients. As soon as AML is diagnosed, AYA patient should be offered to participate in well-designed clinical trials. The standard treatment approach for AYAs with AML is remission induction chemotherapy with an anthracycline/cytarabine combination, followed by either consolidation chemotherapy or stem cell transplantation, depending on the ability of the patient to tolerate intensive treatment and cytogenetic features. Presently, continuing progress of novel drugs targeting specific pathways in acute leukemia may bring AML treatment into a new era.

## 1. Introduction

The adolescent and young adult (AYA) population is defined by an age group range approximately between 15 and 35 years of age [1]. About 70,000 adolescents and young adults (ages 15–39) are diagnosed with cancer each year in the United States.

Acute myeloid leukemia (AML) represents about 33% of adolescent and 50% adult leukemia [2]. In United States, using 1975–2011 US Surveillance Epidemiology and End Results (SEER) data, annual rates for new diagnosis of AML in AYAs are 4.4/100,000, but survival data for the specific age groups of AYA are scarce. Although population-based data from England and Wales 1993–1998 years showed a five-year survival of 46%, while US SEER 1975–2000 years registry data showed a 20-year survival of 20%–27% for 15–29-year-old AYA with AML [2].

The improvement of prognosis in childhood and adolescent AML has been seen over the past two decades; CR with intensive induction therapy 80%–90% and 60%–65% are cured after post remission therapy. Recently, Children Oncology Group (COG) reported the outcomes of 238 AYA patients (16 through 20 years) from four serial childhood COG trials (CCG-2891, CCG-2941, CCG-2961 and AAML03P1) between 1989 and 2006 [3]. In the evaluation of this report, AYA patients were more likely to have poor-risk disease and are less likely to have standard-risk disease. Nevertheless, the outcome of AYA was similar to children patients with newly diagnosed AML. Notably, there are exact differences in treatment-related mortality as higher rates of infection-related deaths were seen in AYAs with AML.

Large prospective studies that include both pediatric and adult patients with AML could not clarify a distinct biology of AYAs [4]. Garrido *et al.* [3] reported that CD34 positive AML stem cells from younger patients express high levels of bcl-2. In contrast to our knowledge, this overexpression reduced the rates of apoptosis, drug resistance and poor clinical outcomes. Cytogenetic is the most important prognosis factor of AML, but only a few studies analyzed the frequencies of genetic abnormalities specifically in AYAs [5,6]. One of the large prospective studies for children and up to age of 55 years old adults, MRC AML 10 trial, established equal cytogenetic group distributions among AYAs aged 14–35 and younger children. The most interesting result of the study was rapid decline of the three-year survival rate from 42% in children to 19% in AYAs [5]. In 2008, Rowley’s review of the cytogenetic of AML mentioned the age-dependent pattern of AML translocations [6]. It was disclosed that *t*(15,17), the characteristic of APL, peaks in incidence between 20 and 39 years of age, and that *t*(11q23), a particularly difficult-to-treat type of AML, has its lowest frequency in this age group [6]. The European Leukemia Net brought out a standardized prognostic scoring system including the conventional cytogenetic and the commonly used molecular testing markers (FLT3-ITD, CEBPA and NPM1) [7]. It is important to note that, no specific genetic or phenotypic aberrations were found in AYAs with AML.

It is clear that in these age groups, patients deserve special attention differences in underlying cancer biology with special medical, physical, psychological and social needs. Unavoidable psychosocial care is the most difficult in the management of adolescents. The need of autonomy and independence, social development, sexual maturation, education and employment generated the conventional problems. In view of these specific adolescents’ needs, it is recommended to treat these patients in special units whenever possible [8].

## 2. Management

### Principles of Treatment

AML treatment in AYA should be based on cytogenetic and molecular factors to avoid overtreatment in patients with favorable prognosis and to improve outcome in those with unfavorable prognosis. Notably, the therapy should be to cure the patient by exterminating the leukemic clone while avoiding side effects and late effects as much as possible. In acute lymphoblastic leukemia, it has been shown that poorer prognosis of AYAs can be overcome with intensive pediatric protocols; whether a similar approach should be applied to AYAs with AML is not evidently provided [2]. Despite several strategies to increase the intensity of therapy, the long-term overall survival rate has not exceeded a plateau of 50%, suggesting that treatment-related toxicity does not balance against antileukemic efficacy [9]. Also, it appears that further intensification of therapy or “one-size-fits-all” treatment strategy will not improve the current AML survival rates.

As soon as AML is diagnosed, AYA patient should be offered to participate into well-designed clinical trials to ensure highest quality and safety of diagnostics and management. Although the majority of children with AML are treated in clinical trials, the number of AYAs enrolled in clinical trials is much lower. Reasons for the poor clinical trial participation in adolescents are undefined differences in biology, poor compliance or intolerance of therapy, receiving care at centers without AYA experience and psychosocial needs [4,8].

In contrast to children and adults, there is no data clarifying the kind of therapy appropriate for AYA. There are also limited prospective and retrospective studies looking into the outcomes for AYAs treated on pediatric *versus* adult protocols. Besides, treatment protocol of AYA depends especially on the individual fitness of the patient and the choice of treating physician or center. As usual, AML treatment consists of induction and consolidation to maintain remission arms. Differing from adults, additional CNS therapy is routine in most of the pediatric protocols [9]. Furthermore, the efficacy of maintenance therapy in AYA has not been proved as with adults and children.

When chemotherapy was introduced into the care of AYA with leukemia, the goal was focused on achieving remission. Long-term complications include effects on reproductive capacity should be separately evaluated. In patients determined to be at risk for future infertility, assisted reproductive technologies such as cryopreservation of gonadal tissue or gametes can be used [10].

## 3. Remission Induction Therapy

The combination of three days of an anthracycline (daunorubicin 60–90 mg/m^2^/day idarubicin 10–12 mg/m^2^/day or mitoxantrone 10–12 mg/m^2^/day) and seven days of cytarabine (100–200 mg/m^2^/day), named as “3 + 7” continues to be the backbone of AML induction therapy over the past four decades [11]. AYA AML patients usually received one or two cycles of induction therapy. On the basis of available evidence in children and adolescents with AML, complete remission (CR) achieved >80% with these regimens.

From 1986 to 2008, Cancer and Leukemia Group B (CALGB) had conducted sequential trials for newly diagnosed AML, named as CALGB 8525, CALGB 9022, CALGB 9222, CALGB 9621 and CALGB 19808. Daunorubicin and cytarabine-based induction treatment were used in all CALGB trials and the CR rate after receiving up to two courses was 76% for the AYA cohort, including 149 patients aged 16–21 years [12]. The summaries of protocols are shown in Table 1.

**Table 1:** Summary of the protocols.

Protocol/Courses (References)	Induction Drugs	Postremission Drugs	BMT
CALGB 9022 [12]	AraC and DNM	HiDAC, VP-16/Cyclophosphamide, and AZQ/mitoxantrone	None
CALGB 9621 [12]	AraC, DNM, VP-16, (±Valspodar)	HiDAC	Autologous (except CBF + AML)
AML BFM-93 [13]	AraC, VP-16, and DNM/Ida	HAM-Consolidation therapy (6-thioguanine; prednisolone; vincristine; adriamycin; Ara-C; intrathecal Ara-C; cyclophosphamide) Intensification (HiDAC and VP-16 and cranial irradiation)-Maintenance therapy (thioguanine and Ara-C)	Allogeneic
MRC AML12 [14]	AraC, VP-16 and DNM/Mitoxantrone (±GCSF)	Amsacrine, AraC, VP-16-AraC, Mitoxantrone/AraC, Ida, VP-16/BMT	Allogeneic Autolog
AML-BFM 2004 [14]	AraC, VP-16 and l-DNM/Ida HAM	AraC/, Ida (±2-chloro-2-deoxyadenosine) HAM-Intensification (HiDAC, VP-16 and cranial irradiation) Maintenance therapy (intrathecal AraC)	Allogeneic
AML 10 [15]	AraC, DNM/Ida/Mitoxantrone	Intermediate-dose AraC	Allogeneic Autolog
MRC AML15 [16]	AraC, DNM, VP-16, Fludarabine, Ida, GCSF (±Mylotarg)	Amsacrine, AraC, VP-16 (±Mylotarg) HiDAC (±Mylotarg)	Allogeneic
PALG [17]	AraC, DNM (±2-chloro-2-deoxyadenosine) CLAG	HAM-HiDAC (±2-chloro-2-deoxyadenosine) Maintenance therapy (AraC, DNM, 6-thioguanine)	Allogeneic Autolog
SWOG S0106 [18]	AraC, DNM, and GO	HiDAC (±GO)	Allogeneic
CALGB 8525 [19]	AraC and DNM	AraC	None
CCG-2891 [20]	Dexamethasone, AraC, 6-Thioguanine, VP-16, DNM, intrathecal AraC	AraC and l-asparaginase-AraC, 6-thioguanine, vincristine, l-asparaginase cyclophosphamide and 5-azacytidine AraC, DNM, VP-16, 6-thioguanine and dexamethasone	Allogeneic Autolog

Abbreviations: AraC, Cytarabine; BMT, bone marrow transplantation; DNM, daunomycin; AZQ, 5 Azacytidine; HiDAC, high dose cytarabine; VP-16, etoposide; Ida, Idarubicin; GO, gemtuzumab + ozogamycin; CBF, Core Binding Factor; HAM, high dose cytarabine and mitoxantrone; G-CSF, granulocyte colony-stimulating factor; CLAG, 2-chloro-2-deoxyadenosin, cytarabine and granulocyte colony-stimulating factor.

Various trials have sought that intensifying induction therapy may improve the initial response rate and the long-term outcome among AML patients. Sequential studies by the Eastern Cooperative Oncology Group (ECOG) first postulated that increasing the dose of daunorubicin 45 to 60 mg/m^2^/day for three days resulted in a median complete remission rate of 62% [21]. Furthermore, same group demonstrated the improvements in rates of complete remission and the overall survival with the administration of 90 mg/m^2^/day for three days of daunorubicin among young adults [22]. Also, another anthracyclin, idarubicin, has been used in AML treatment since 1980s. The AML-BFM 93 trial evaluated the efficacy of idarubicin (12 mg/m^2^/day × 3 days) compared with daunorubicin (60 mg/m^2^/day × 3 days) in AML induction therapy in children and adolescent. Individuals in idarubicin arm improved blast cell clearance (<5% blasts on day 15); higher CR rates were found in younger patients without an overall survival benefit [13]. The similar results were also obtained in subsequent studies. The anthraquinone derivative mitoxantrone has also been commonly used as part of effective induction regimens for a long time. In the United Kingdom, Medical Research Council Acute Myeloid Leukemia 12 (MRC AML12) trial comprising 1243 AML patients (between 15 to 59 years old) received cytarabine (100 mg/m^2^/day × 10 days), etoposide (100 mg/m^2^/day × 5 days) and either daunorubicin (50 mg/m^2^/day × 3 days) or mitoxantrone (12 mg/m^2^/day × 3 days) for induction; the results showed no survival benefit with comparable acute toxicity [14]. After these results, the children subgroup analysis of the overall study published revealed significant reduction in relapse with mitoxantrone, but due to higher rates of treatment related mortality, this did not turn out to be a significant improvement in overall survival [23]. It is noteworthy that in the EORTC and GIMEMA Groups Study AML-10 trial, one of the largest study in AML aged 15–60 years, where patients were randomly assigned to daunorubicin 50 mg/m^2^/day, mitoxantrone 12 mg/m^2^/day or idarubicin 10 mg/m^2^/day (each given with cytrabine and etoposide) on days one, three, five and after one or two courses of induction therapy, similar outcomes were observed between the treatment arms [15]. There is enough evidence to indicate that intensification of daunorubicin may have a beneficial effect without increasing toxicity and that the choice of anthracycline varies depending on the current conditions [24].

It is important to note that the most feared adverse effect of anthracyclines is acute and late cardiotoxicity. Several reports have already reported that children and adolescents are more comfortable with anthracycline cardiotoxicity depending on continuous heart muscle growth. After liposomal encapsulation of daunorubicin was discovered as they consume higher doses without increasing cardiotoxicity. Interestingly, AML-BFM 2004 trial reported that liposomal daunorubicin (80 mg/m^2^/day × 3 days) and standard induction with idarubicin (2 mg/m^2^/day × 3 days) had similar response rates after induction with comparable early and late cardiotoxicity among <18 years old pediatric and adolescent population [24]. Nevertheless, in patients with cardiac disease, liposomal daunorubicin should be used.

Also, different leukemia groups studied cytarabine dose escalation. The randomized comparison between high dose (200 mg/m^2^/day; days 1–10 every 12 h) and standard dose (100 mg/m^2^/day; days 1–10 every 12 h) cytarabine displayed similar outcomes (overall response was 84% *vs.* 85%; eight-year survival was 31% *vs.* 32%) in the MRC AML12 trial [14]. In subgroup analysis of 708 children (2–15 years old) and 541 AYA (16–24 years old) patients, 10 year OS and relapse rates were found to be 47% *vs.* 59% and 47% *vs.* 42% (*p* > 0.05), respectively [4]. Evaluation of high-dose cytarabine (HiDAC) combined with daunorubicin in induction has been reported by different studies; the Southwest Oncology Group (SWOG; 2 g/m^2^ bid × 6 days), the Australian Leukemia Study Group (ALSG; 3 g/m^2^ bid on days one, three, five, and seven) and the Eastern Cooperative Oncology Group (ECOG; 3 g/m^2^ on days one, three, and five) [6]. Neither of these trials revealed a higher CR rate with HiDAC nor observed increased toxicity in all. It is important to note that the administration of HiDAC in induction regimens has failed to improve the outcome and thus cannot be recommended outside clinical trials.

A third drug (e.g., etoposide or 6-thioguanine (6-TG)) is usually added in induction of pediatric protocols even if conducted studies failed to demonstrate any therapeutic benefit from addition of 6-TG or etoposide to “3 + 7”. Between 2002 and 2009, 385 patients aged 15–29 years were enrolled on the MRC AML15 trial; the addition of etoposide to “3 + 7” showed no benefit in rate or durability of response (ORR of all cohort: 86% *vs.* 84%) [16]. Moreover, CALGB 7921 trial suggested that instead of adding 6-TG, cytarabine and daunorubicin dose or cycle intervals could be edited [25].

Other drugs added to standard induction treatment of AYAs include: A purine analog such as fludarabine or cladribine and a calicheamicin conjugated to a CD33 antibody as gemtuzumab ozogamicin (GO). Polish Adult Leukemia Group (PALG) have evaluated cladribine or fludaribine added to daunorubicin and cytarabine. They found 12% increase in three-year-OS and 10 months extension of the median survival in cladribine arm compared with the standard induction arm. Furthermore, treatment related toxicity was comparable in both arms [17]. In addition, same study group reported similar survival outcomes between fludarabine and the standard induction arms [26]. As a result of these studies, cladribine added regimen as remission induction should be considered as a new treatment option, but fludarabine addition seemed not to have any advantage. In 2010, the results of the SWOG S0106 trial has been shown that GO decreased CR rates with higher mortality among children and adolescent and hence, withdrawn [18]. Consequently, the recently published AAML0531 trial sought the efficacy of GO added induction therapy and showed improvement in EFS but not OS among children and adolescent through reduced relapse rate. Although treatment related mortality was increased in GO added arm, in spite of similar overall toxicity incidence between both arms [27]. Future investigation is required to optimize the administration of GO.

Interestingly, several reports have already suggested that after the first administration of cytotoxic agents in AML, leukemia cells can be recruited into the cell cycle and then become more sensitive to cell cycle-specific agent 6 to 10 days after the initial chemotherapy exposure. The Children Cancer Group (CCG) conducted four trials; each used an “intensive-timing” induction regimen. In the CCG-2891 trial, pediatric and adolescent AML patients received a four-day cycle of five-drug induction therapy (dexamethasone, cytrabine, 6-thioguanine, etoposide and daunorubicin (DCTER)); cycles are repeated either every 10 days (intensive-timing) regardless of low blood counts and side effects of the treatment or every 14 days (standard-timing) depending on bone marrow recovery [20]. Although AYAs treated on this CCG protocol had higher incidence of remission (74% in all population), 10 year OS was 45% ± 6% because of the higher rate of treatment related mortality (26% ± 6%) [20]. Altogether, these results and the aforementioned study indicate that pediatric protocols would be better for AYAs if the treatment related mortality with these regimens could be reduced.

Recently, Woods *et al.* reported the comparison results between pediatric and adult protocols in 517 AYAs with AML using data from three large cooperative groups (COG, CALGB, and SWOG) and demonstrated that aggressive pediatric protocols provide more anti-leukemia efficacy with halve of relapse rates but higher treatment related-mortality. Ten year OS was higher for the COG AYA cohort than for the other two adult cohorts (45% *vs.* 34%, *p* = 0.026) [12]. To our knowledge, there has been no approach beyond 1–2 course in 7–10 days cytarabine and three days of an anthracycline combination for induction treatment in AYAs with AML.

## 4. Postremission Therapy

Pretreatment karyotype and bone marrow assessment on the 7th or 10th day after completion of induction treatment determine the type of post-remission therapy. Many studies, herein mentioned used HiDAC or allogeneic hematopoietic stem cell transplantation (HSCT) as post-induction treatment.

Intensification of consolidation is as important as intensive induction therapy; since 1980s, the CALGB study group has conducted various trials to find the best intensive post-remission therapy for younger AML patients. In 1994, the results of CALGB 8525 trial was published and four courses of HiDAC (3 g/m^2^ per every 12 hours on days one, three, and five) was found to be superior to lower/intermediate-dose cytarabine (either 100 or 400 mg/m^2^/day on days 1–5) in young adults [19]. In addition, subsequent studies has shown that HiDAC improved survival rates in patients with Core Binding Factor (CBF)-AML more than Cytogenetically Normal (CN)-AML (five years OS; 64% *vs.* 35%) [28]. Also in 2008, the German-Austrian AML Study Group (AMLSG) reported the efficacy of HiDAC in AML with mutated NPM1 without FLT3-ITD and with mutated CEBPA [29].

NOPHO AML 93, MRC AML 10, AML-BFM 2004 and AML99 trials has been shown to decrease the relapse rates with intensive induction protocols that include HiDAC in pediatric population [30]. Unlike adult studies, non-cross-resistant drugs used in induction are mostly administered a total of 2–5 courses as consolidation and maintenance treatment in children and adolescent with AML. However MRC AML-12 trial has revealed that more than four courses of post-remission treatment did not improve the outcomes [14].

In MRC AML15 trial, young adults alive after two courses of induction therapy were randomly allocated either MRC consolidation (amsacrine, cytarabine, etoposide, and then mitoxantrone and cytarabine (MACE-MidAC)) or the international standard consolidation (HiDAC). The results of the study showed similar outcomes with increased treatment related to hematologic toxicity in MACE-MidAC arm. According to the evaluation of cytogenetic risk group, OS for poor-risk patients determined was significantly higher in MRC consolidation arm; the group concluded that one course of MACE-MidAC is a superior consolidation option for high-risk AML patients who are not eligible for HSCT [16].

The German Acute Myeloid Leukemia Cooperative Group (AMLCG) investigated the effectiveness of maintenance treatment in younger AML since 1985. In 2003, they postulated the comparison of maintenance treatment (monthly courses of cytarabine 100 mg/m^2^ every 12 h for five days, different second drug every course, daunorubicin, 45 mg/m^2^/day on days three and four (course 1), 6-thioguanine 100 mg/m^2^ every 12 h on days one to five (course 2), cyclophosphamide 1 g/m^2^/day on day three (course 3), 6-thioguanine again (course 4)) between intensive consolidation treatment (cytarabine 1 g/m^2^ every 12 h on days one, two, eight, and nine) [31]. The aforementioned trial has revealed equal CR rates between both arms even though relapse free survival was higher in poor-risk AML patients who received maintenance treatment. A group from MD Anderson Cancer Center used azacitidine (32 mg/m^2^/day for five days in each of four 30-day) after HSCT as a new remission consolidation/maintenance protocol in high risk AML [32]. They established that azacitidine might improve EFS and OS with acceptable toxicities. Recently, azacitidine combined with lenalidomid (azacitidine 50–75 mg/m^2^/day for five days, lenalidomide 5–10 mg/day on days 5 to 25) was administered to 10 patients with AML in remission after induction as maintenance treatment and provided the immunological benefits of this regimen [33]. It is important to note that maintenance treatment is not a standard part of AML treatment and still need further investigations.

Developments in transplantation since 1980s have led to increasing improvements in survival and reduction of treatment-related mortality (TRM) among HSCT recipients. Therefore, HSCT as frontline post induction therapy is recommended for all aged patients with intermediate-unfavorable risk AML even if HSCT in low-risk group during first remission is unknown in children and young adults. Surprisingly, the results from MRC AML10 and AML12 trials showed no survival benefit with allo-HSCT in first CR for any risk group in AYAs [14,34]. They concluded that favorable-risk patients should not undergo transplantation in first CR, but children and adolescent with intermediate-high risk should be weighed on case-by-case basis. It has already been known that SCT from HLA-matched (8/8 or 10/10 allele matched) related donor is more beneficial than continued chemotherapy; the important advantage in preventing relapse may be offset by high rates of transplant-related deaths because of the wrong optimal timing.

In 2012, the CIBMTR published the outcomes of AYAs with AML treated with HLA-identical sibling (MSD) or unrelated (URD) HSCT between 1980 and 2005. They reported improvement of five years OS and reduction of transplant-related mortality (TRM) from 1998 to 2005, compared with 1980 to 1988 (Due to MSD data OS at five years: 43% *vs.* 37%, *p* > 0.05; TRM: 39% *vs.* 20%, *p* = 0.01) [35]. This can be explained with continued improvements in AML prognostic factors, HSCT conditioning regimens, HLA typing and HSCT supportive care.

Pediatric cooperative groups conducted various trials that compared allo-SCT with intensive consolidation chemotherapy in children and adolescents with newly diagnosed AML. In 2008, COG published a meta-analysis of four cooperative group studies (Pediatric Oncology Group (POG) 8821, Children’s Cancer Group (CCG) 2891, CCG 2961, and Medical Research Council (MRC) 10), which used HLA matched related allo-HSCT in first remission for children and adolescent patients. The results of these analyses showed the improvement of outcomes in favorable-risk AML patients, but the point to be considered is that only 2/3 of the patients have their risk stratification and cytogenetic results [36]. The MRC 10 and 12 trials revealed better outcomes in children than in AYAs; therefore, the survival benefit could not be shown in both groups who underwent allo-HSCT [14,34]. Despite these results, the beneficial effect of allo-HSCT has been proven in severally randomized young adults trials compared to other approaches.

Although there is no consensus on optimal conditioning regimens for AYAs, most centers use non-TBI-containing myeloablative protocols (MAC) first or subsequent remission. Busulfan and cyclophosphamide are the most administered drugs [37]. Also, treosulphan and fludarabine containing reduced intensity conditioning regimens (RIC) have recently begun to be used with minimal TRM, but still need more prospective trials for recommendation [38]. There is a recently terminated prospective randomized Bone Marrow Transplantation-Clinical Trials Network’s BMT-CTN 0901 clinical trial (NCT01339910) comparing RIC regimens with MAC in AML/MDS; the results explain the optimized conditioning intensity. TBI is no longer preferred because of its late effects and increased risk of seconder malignancies [30]. Notably, TBI did not improve EFS during first remission in some of the pediatric and young adult AML trials [39,40]; however, better EFS with higher doses of TBI was found among AYAs in a review of the literature [41]. Recently, different cooperative groups are seeking the effectiveness of adding fractionated TBI to conditioning regimens in selected advanced patients with AML [42].

Since AYAs with high-risk AML who lack a matched sibling donor (approximately 70%–75% of patients with AML) may benefit from alternative approaches including matched unrelated (including cord blood products) or mismatched family donor, decision should be made based on the urgency of the HSCT [43].

Recently, Kelly *et al.* compared the clinical outcomes after MRD or MUD HSCT during first remission in a total of 159 children (74 of them were AYAs) with high-risk AML using data from COG trials (CCG 2891, POG 9421, CCG 2961, and AAML03P1) and CIBMTR reports. Their results demonstrated similar OS and TRM between both arms with lower relapse risk after only MUD HSCT in a competing risks regression model (HR 0.43, *p* < 0.01) when compared to post-remission chemotherapy [44]. Various studies have also revealed no superiority between MRD and MUD HSCT outcomes (relapse, GVHD and survival) of young adults with CN-AML. However, the most limiting factor is the time taken (median four months in most countries) for the identification of a suitably matched unrelated donor and unfortunately, patients may relapse during this waiting period.

Ebihara *et al.* reported the clinical results of 16 AYA patients who underwent unrelated cord blood transplantation (CBT) during 1999 to 2009. All patients received myeloablative-conditioning regimen and the OS and the DFS at three years were determined to be 67.5% and 48.6%, respectively, a comparable published result of adults and children with AML [45]. During 1996 to 2010, 67 adolescent (38 treated with UCB and 29 treated with Haploidentical sibling SCT) diagnosed with acute leukemia were enrolled in a retrospective study from Spain. The overall outcome including leukemia free survival was better in haplo-SCT arm; TRM was higher in UCB-SCT arm and unexpectedly, the cumulative incidence of relapse was similar in both arms [46]. To our knowledge, the most important factor that restricts the widespread use of CBT is the amount of CD34+ stem cells from the individual graft; though double cord transplant was done to overcome this limitation. The ongoing BMT-CTN 1101 trial (NCT01745913) clarifies the optimal alternative donor source by comparing double UCB with haploidentical transplant.

Haploidentical SCT recently became an effective treatment option in the AYAs with AML candidate for HSCT with lacking an HLA-identical donor or with urgent need to perform SCT. Since every individual has, at least, one mutual HLA haplotype with each biological parent or child, then a suitable donor can be identified within days. In 1998, the Perugia group found leukemia free survival rates of 45%–50% and DFS rates of 30%–45% for young adults with AML receiving transplantation of bone marrow from related one HLA haplotype identical donor. TBI with myeloablative conditioning regimen was admitted and the product consist of large numbers of T-cell-depleted hematopoietic stem cells [47]. Consequently, slowed immune reconstruction, higher rates of TRM and stimulation of graft-*versus*-leukemia effector mechanism could be seen in this kind of transplant. Data from the EBMT registry showed that leukemia free survival at two years was 48% for patients in first remission; however, for patients in non-remission, this rate dropped to 1% [48]. Based on these results, it may be considered that haplo-SCT has more anti-leukemia effect in the early phase of high-risk AML treatment with increase risk of GVHD and graft rejection. Luznik *et al.* developed a new approach to reduce haplo-HSCT related toxicity; post-transplant cyclophosphamide was added to a marrow graft after reduced intensity conditioning regimen [49]. The approach decreased the cumulative incidence of severe GVHD and TRM. Further investigations are required to recommend haplo-HSCT for AYAs with AML in first remission.

Autologous HSCT during the first remission has been commonly used for the past two decades as post remission treatment in all aged patients with high risk AML who are not eligible for MRD HSCT. However, the results of studies comparing auto-HSCT with the other post remission approaches are conflicting. MRC AML10 trial showed the improvement in DFS with similar OS; although CCG 2891 trial has reported that auto-HSCT in CR1 did not have better impact on outcomes compared to post induction chemotherapy [34,50]. It has to be mentioned that these different trials have not used the same intensive induction treatments before auto-HSCT. When we looked into analyses of MRC AML10 trial comparing autologous *versus* allogeneic HSCT, we found that allo-HSCT has improved the survival rates and reduced the relapse rates [34]. Also, the EORTC-LG/GIMEMA AML-10 trial has been determined that younger or high/very high-risk AML patients had better outcome with allo-HSCT compared with auto-HSCT [51]. Altogether, these results showed that auto-HSCT has failed to improve the outcome among young adults population and cannot be recommended for AYAs with AML in first CR outside clinical trials [52].

## 5. Primary Refractory and Relapsed Acute Myeloid Leukemia

For acute myeloid leukemia (AML) patients in complete remission, maintaining remission is a necessity. To our knowledge, the prognosis of relapse depends on the molecular profile of leukemia, the duration of first CR, the age of patient and the type of post-remission therapy administered. Moreover, with the current intensive protocols, up to 20%–30% of young and 40%–50% of older AML patients experienced primary induction failure [53]. More than 50% of the patients in complete remission relapsed within one year (except for AYAs with favorable risk of cytogenetics); relapsed/refractory AML patients have a very poor outcome with 10% of survival incidence. Despite the knowledge of leukemia pathophysiology, the prognosis following relapse is still uniformly poor. MRC AML10 trial reported the median relapse time to be 10 months and the OS after relapse to be <40% in children and AYAs [34].

For younger patients with relapsed/refractory AML, the enrollment in a clinical trial should be the first approach. If there is a late relapse patient, retreatment with the previously used induction regimen should be employed. Notably, if the relapse is detected at the early phase of tumor burden, HSCT should be considered after salvage chemotherapy. Since there are few prospective controlled studies assessing different treatments, thus no standard salvage chemotherapy can be recommended.

In relapsed/refractory AML, the aim was to achieve second remission using various protocols; such as induction therapy antimetabolite (cytarabine, fludarabine) and anthracycline-based. HiDAC is widely used for induction treatment of relapsed/refractory AML. In 2012, Larson *et al.* published the outcomes of HiDAC/mitoxantrone in younger adults with relapsed or refractory high-risk AML and reported an overall response rate of 55% with the induction death rate of 9% [54]. The Japanese Childhood AML Cooperative Study Group AML99 trial determined the results of children and adolescent with relapsed AML patients who received treatment protocols including; cytarabine, etoposide and idarubicin/mitoxantrone [55]. The study showed that the second remission with cytarabine based reinduction regimens was 50% and that the five-year OS was 37%.

To evaluate the CR rates of refractory/relapsed AML in younger adults, the Japanese Adult Leukemia Study Group (JALSG) conducted a phase II study of FLAGM (fludarabine 15 mg/m^2^ every 12 h on days 1–4, Ara-C 2 g/m^2^ on days 1–4), G-CSF 300 μg/m^2^ on days 1–4, and mitoxantrone 10 mg/m^2^ on days 3–5 protocol [56]. Seventy percent response rate was provided by FLAGM in relapsed or refractory AML patients; furthermore, randomized studies are still warranted to confirm this option.

In the study of Relapsed AML01/2001, 394 children (younger than 21 years) were randomized equally for FLAG (Fludarabine 30 mg/m^2^/day, on days 1–5, Ara-C 2 g/m^2^/day, on days 1–5, and G-CSF 200 μg/m^2^ on days 0–5) *versus* liposomal daunorubicine (60 mg/m^2^/day, on days 1, 3 and 5) + FLAG. Liposomal daunorubicine (DNX) added FLAG significantly improved the early response from 70% to 80% and CR rate from 70% to 80%. However, the OS was similar between two groups [57].

Malfuson *et al.* published a small retrospective analysis of the outcomes for 14 young adults with first relapsed AML; those who received 3 + 7 + GO induction regimen [58]. The overall response rate was reported to be 79% with a tolerable toxicity profile. GO + FLAG (Fludarabine 30 mg/m^2^/day, on days 1–5, Ara-C 2 g/m^2^/day, on days 1–5, GO 3 mg/m^2^/day on day 1, and G-CSF 3 mg/kg/day) also studied in relapsed/refractory AML reported excellent clinical and molecular response in 29 of the 34 young adults with CBF-AML [59]. The major restriction of GO studies is the number of patients (small population); large, randomized and prospective trials would be required to recommend first relapsed.

In first relapse, all type of HSCT is widely preferred approach after achieving a second remission. For patients who are not eligible for allo-HSCT in second remission, auto-HSCT can be considered. Auto-HSCT data from the British Society of Blood and Marrow Transplantation (BSBMT) registry has shown five-year OS of 32% and disease-free probabilities of 30%–35% [60]. Notably, most relapses were detected within the first 24 months of auto-HSCT.

Allogeneic HSCT from HLA-matched or mismatched donor is the most effective consolidation therapy once a new remission has been obtained. However, most of the studies in relapsed pediatric AML have demonstrated poor outcome except those patients with late relapse and favorable AML. Recently, the International BFM-Study Group has shown 38% overall survival for allo-HSCT in children and adolescent with second remission [57]. RIC was commonly offered before allo-HSCT in relapsed patients depending on the age of the patient, the poor performance status, the co-morbidities and the prior treatments with lesser TRM and more GVL effect.

## 6. New Therapy Approaches

To the best of our knowledge, the treatment of AML was based on 1–2 course remission induction chemotherapy, followed by either consolidation therapy or HSCT depending on the prognostic features of the patients. However, enormous progress in the understanding of AML pathogenesis and improvements in molecular genomic technologies are leading to novel target agents and the development of personalized and risk-adapted treatment.

Clofarabine (2-chloro-2′-fluoro-deoxy-9-b-d-arabinofura-nosyladenine) is a second-generation nucleoside analog, which inhibits both ribonucleotide reductase and DNA polymerase. Investigators from the MD Anderson Cancer Center (MDACC) conducted various trials with single agent clofarabine or combined with cytarabine and/or idarubicin; a favorable outcome was derived even in adults with primary refractory or relapsed AML [61]. In 2009, Hijiya *et al.* published the result of clofarabine, cyclophosphamide and etoposide combined regimen in five pediatric (between age 5–21 years) patients with AML and 100% OR (1 CR and 4 CR in the absence of total platelet recovery (CRp)) [62]; this highly promising response reported in this small population warrants further study. To our knowledge there is no published trials and only one ongoing COG trial (clinicaltrials-gov #NCT00372619) to clarify the benefit of clofarabine and cytarabine combination in 1–30 years old patients with refractory or relapsed acute myeloid leukemia.

The hypomethylating agents; azacitidine and decitabine have shown single agent benefit in AML. There are various ongoing trials for azacitidine either as single or combined agent (clinicaltrials-gov #NCT01839240, #NCT01249430, #NCT01861002, #NCT00422890). In the French ATU retrospective trial, 11% response rate was found among patients with refractory or relapsed AML treated with azacytidine [56]. It seems that single agent azacytidine has only limited efficacy in relapsed or refractory AML. In a phase I study, 23 patients with relapsed or refractory AML were treated with the combination of 5-azacytidine and bortezomib; the results of the trial showed 26% response rate. Recently, Phillips *et al.* showed the efficacy of low dose decitabine (20 mg/m^2^/day 10 days) in eight children and young adults with heavily pretreated relapsed/refractory AML and reported only 38% of the patients achieved CR with very favorable toxicity profile [63].

Internal tandem duplication mutation of FMS-like tyrosine kinase 3 (FLT3-ITD mutations) was found in 20% adult and 15% pediatric AML patients associated with poor prognosis [64]. The patients are candidates for targeted therapy; although several FLT3-inhibitors (sorafenib, crelonatinib, quizartinib, midoustaurin) have been studied on various trials, herein mentioned some milestone trials in young adults.

Several groups have shown that Sorafenib, a multikinase (FLT3, c-KIT, NRAS, and Raf kinas) inhibitor, has impressive response and a tolerable safety profile in adults with AML as a single agent or in sorafenib-containing induction protocol. In SORAML trial, 264 younger patients were randomized to achieve sorafenib (800 mg/day during induction, consolidation and 12 months maintenance period) or plasebo adding 3 + 7 (cytarabine 100 mg/m^2^/day 7 days, daunorubicin 60 mg/m^2^/day 3 days) and following HiDAC as consolidation. The results of the study CR rates and 2 year OS were higher in sorafenib arm (sorafenib *vs.* placebo; CR: 60% *vs.* 56%, *p* = 0.62; OS: 66% *vs.* 72%, *p* = 0.37) with increased incidence of treatment related toxicity [65]. In a phase I study, 11 children (aged between 6–17 years) with acute myeloid leukemia were treated with sorafenib (200 mg/m^2^ and 150 mg/m^2^ twice daily, with maximum doses of 400 mg and 300 mg; alone on days one to seven and 13 to 28), clofarabine (40 mg/m^2^ on days 8 to 12), and cytarabine (1 g/m^2^ on days 8 to 12). Leukemia response was evaluated on day eight and seven patients (63%) revealed more than 50% reduction of baseline bone marrow blasts regardless of FLT3 status [66]. In summary, the benefit of sorafenib was proven in different, prospective, randomized trials for treatment of newly diagnosed or relapsed/refractory AML. Futher prospective studies on sorafenib would be required to confirm all the stated results.

Lestaurtinib is a potent inhibitor of FLT3 and triggers apoptosis in FLT3-ITD leukemic blasts. The Cephalon 204 trial patients (aged between 18 years and older) with AML were randomized either with chemotherapy (MEC/HiDAC) or lestaurtinib (80 mg/day every 12 h, beginning two days after the completion of chemotherapy (day seven)). Lestaurtinib failed to improve the outcomes; however, only 58% of the patients in lestaurtinib arm reached LT3 inhibition; as estimated from the PIA assay, on day 15 and those improved response rate and OS [67].

Midostaurin (PKC412) is an inhibitor of FLT3 tyrosine kinase, VEGFR, PDGFR, and c-KIT. In a phase I trial of midostaurin, 35% and 25% of the patients show reduction in the amount of peripheral and bone marrow blasts [68]. The ongoing CALGB study clarifies the addition of midostaurin to induction, consolidation therapy and followed single agent midostaurin as maintenance treatment as frontline therapy of younger adult patients with AML (ClinicalTrials.gov: NCT00651261).

In conclusion, with the continued progress of novel drugs targeting specific pathways in acute leukemia, AML treatment may be brought into a new era. It is important to note that more clinical trials should be conducted in order to understand the benefit of targeted agents in AYA population.

## 7. Conclusions

Age is an independent prognostic factor in AML; the prognosis of AYAs diagnosed with AML is better than those in young adult patients. However, there were no genetic or phenotypic abnormalities reported on AML in AYAs. Treating AYA patients with AML is still challenging since there is no data clarifying the kind of relevant therapy for AYA. Based on limited prospective and retrospective AYA studies, a prospect for cure of about 50% was achieved whether treated on an adult or pediatric protocol. We therefore suggest that encouraging AYAs to participate in clinical trials is the only way to improve response rate.
